# Retroperitoneal smooth muscle tumor of uncertain malignant potential after hysterectomy: a case report

**DOI:** 10.1186/1752-1947-5-214

**Published:** 2011-05-28

**Authors:** Hye Sung Won, Hoo Geun Chun, Kyungji Lee

**Affiliations:** 1 Division of Medical Oncology, Department of Internal Medicine, Uijeongbu St Mary's Hospital, Kyonggi-do, Korea; 2 Division of Medical Oncology, Department of Internal Medicine, Seoul St Mary's Hospital, Seoul, Korea; 3 Department of Hospital Pathology, Seoul St Mary's Hospital, Seoul, Korea

## Abstract

**Introduction:**

Smooth muscle tumors of uncertain malignant potential represent a histologically heterogeneous group of uterine smooth muscle tumors that cannot be diagnosed as either benign or malignant. Smooth muscle tumors of uncertain malignant potential are usually clinically benign, but should be considered tumors of low malignant potential because they can occasionally recur or metastasize to distant sites.

**Case presentation:**

We report the case of a 62-year-old Mongol woman diagnosed with a retroperitoneal smooth muscle tumor of uncertain malignant potential and lung metastasis, with a history of prior hysterectomy. The case was initially misdiagnosed as retroperitoneal sarcoma, and our patient received chemotherapy. However, no interval change in the size of the retroperitoneal mass and metastatic lung nodules was seen over a period of at least five years. She underwent partial resection of the retroperitoneal mass for the purposes of debulking and establishing a histopathological diagnosis. The diagnosis of the retroperitoneal mass was then confirmed as a smooth muscle tumor of uncertain malignant potential.

**Conclusion:**

Smooth muscle tumors of uncertain malignant potential have an unpredictable clinical course, and relapses generally appear to occur after a long disease-free interval of up to several years. Therefore, patients diagnosed with smooth muscle tumors of uncertain malignant potential should receive long-term follow-up.

## Introduction

Smooth muscle tumors are histologically categorized into leiomyomas and leiomyosarcomas, based on the combination of histological parameters such as mitotic activity, cytological atypia and coagulative tumor cell necrosis [1]. Smooth muscle tumors of uncertain malignant potential (STUMPs) represent a histologically heterogeneous group of uterine smooth muscle tumors that do not clearly fall into the category of either leiomyomas or leiomyosarcomas [2]. The clinical behavior of STUMPs is poorly understood. In previous reports in the literature, most uterine STUMPs had a benign clinical course and were successfully treated with myomectomy or hysterectomy. We report the case of a 62-year-old woman who presented to our facility with a retroperitoneal STUMP with lung metastasis, eight years after a hysterectomy.

## Case presentation

A 62-year-old Mongol woman was referred to our hospital for further evaluation of an intra-abdominal mass seen on an abdominal computed tomography (CT) scan. She had visited her primary care physician for recently aggravated chronic constipation and intermittent abdominal discomfort. These symptoms had persisted for more than 20 years. Whenever these symptoms were exacerbated, she took medications such as painkillers and laxatives prescribed by several private hospitals. She had no other gastrointestinal symptoms, and there was no change in body weight. Additionally, there were no respiratory or cardiovascular symptoms.

Her medical history was significant for the following illnesses. In 1994, she attended hospital for intermittent lower abdominal pain and constipation; on this occasion she was found to have a large retroperitoneal mass on an abdominal CT scan. Additionally, a chest CT scan showed small, well defined nodular lesions in both peripheral lung fields. A sonography-guided fine needle aspiration biopsy of the retroperitoneal mass was performed. The cytological findings revealed an atypical spindle cell tumor, and differential diagnoses of malignant fibrous histiocytoma, leiomyosarcoma, fibrosarcoma and malignant schwannoma were considered. At that time, she was diagnosed with retroperitoneal sarcoma with lung metastases. She received six cycles of ifosfamide (1440 mg/m^2 ^on days one to five) and etoposide (80 mg/m^2 ^on days one to five). After completion of the planned chemotherapy, there were no significant interval changes in the size of the retroperitoneal mass. Additionally, she received six cycles of CYVADIC (cyclophosphamide 500 mg/m^2^, doxorubicin 50 mg/m^2^, vincristine 1.5 mg/m^2 ^on day one, dacarbazine 250 mg/m^2 ^on days one to five) chemotherapy. She was then lost to follow-up and had received no further treatment since then.

In November 2005 and January 2007, she visited the Department of Respiratory Medicine at our hospital due to symptoms of upper respiratory infection. She underwent abdominal and chest CT scans on both these visits. Between 2005 and 2007, her CT findings showed no significant change in the size of the retroperitoneal mass and lung metastatic nodules. At eight years before the diagnosis of retroperitoneal sarcoma was established, she had undergone a total hysterectomy for uterine leiomyoma at Korea Cancer Center Hospital, Seoul, Korea. Apart from these incidents, she had no other medical problems and her family history was unremarkable. She had no history of alcohol use or smoking.

**Figure 1:**
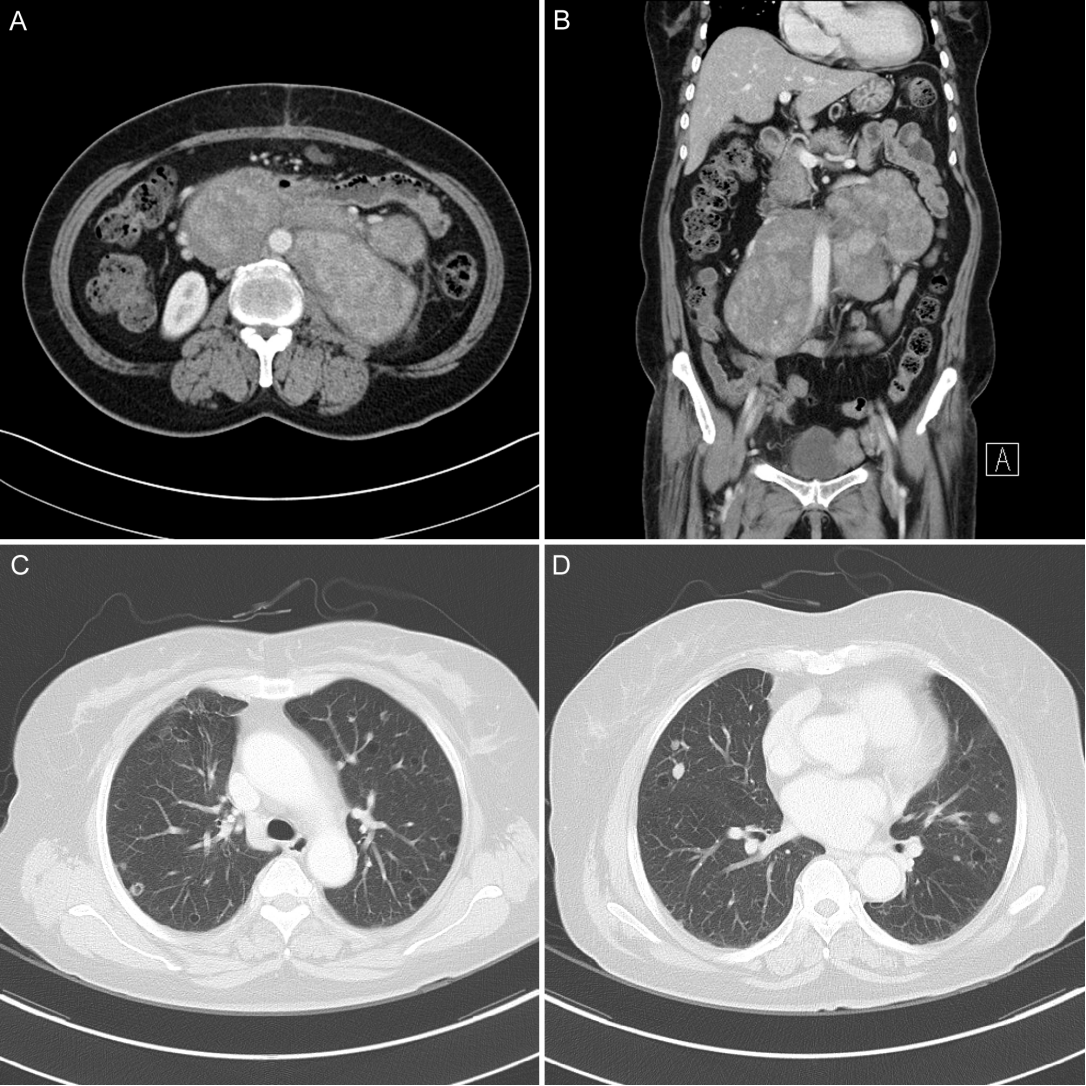
**Computed tomography (CT) scan results**. (a, b) Abdominal CT scan showing 8.8 × 16.7 cm sized multilobulated, well defined enhanced masses in retroperitoneal space. (c, d) Chest CT scan showing multiple tiny to small solid, cavitary metastatic nodules in both lungs.

**Figure 2:**
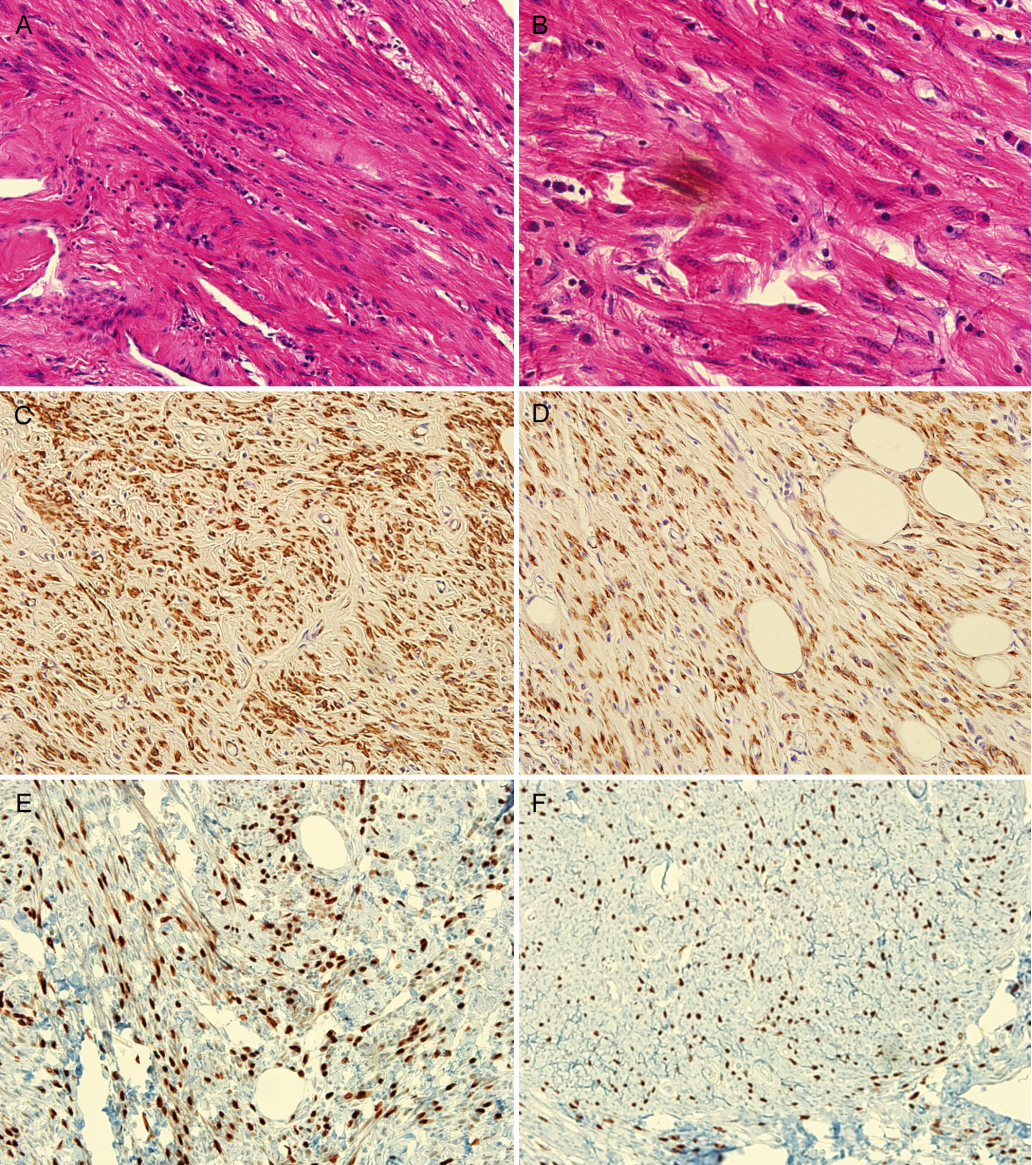
**Histological appearance of the retroperitoneal smooth muscle tumor of uncertain malignant potential (STUMP)**. Spindle cell proliferation ((a) hematoxylin and eosin, 200 ×) with mild to moderate nuclear atypia ((b) hematoxylin and eosin, 400 ×). (c, d) Immunohistochemical staining for actin and desmin (200 ×). (e, f) Immunohistochemical staining for estrogen receptor (ER), and progesterone receptor (PR) (200 ×).

On physical examination, her breath sounds were clear and there were no palpable lymph nodes. Her abdomen was soft and obese, and there was no abdominal tenderness on palpation. No abdominal masses were identified on palpation, and there was no organomegaly. Her blood pressure was 140/90 mmHg, hemoglobin concentration was 15.6 g/dL, white blood cell count was 7600 cells/μL, and platelet count was 205,000 cells/μL. Her serum lactate dehydrogenase (LDH) level was mildly increased to 564 U/L. Other laboratory findings were within their normal ranges. Serum levels of tumor markers cancer antigen (CA) 125, CA 19-9, and carcinoembryonic antigen (CEA) were normal. An abdominal CT scan showed multiple large, well defined, highly enhanced masses in the retroperitoneum and pelvic cavity, and there were no significant changes in the sizes of the masses since November 2005 (Figure 1). A chest CT scan showed multiple, tiny to small cavitary and cystic metastatic nodules in both lungs, and there was also no interval change since November 2005 (Figure 1). There was no significant mediastinal or hilar lymphadenopathy. A F-18 fluorodeoxyglucose positron emission tomography/computed tomography (F-18 FDG PET/CT) scan showed conglomerated bulky masses with inhomogeneous FDG uptake (maximum standard uptake value (SUVmax) of 4.0) in the retroperitoneum and pelvic cavity. We planned to perform a CT-guided percutaneous lung biopsy, but her pulmonary lesions were too small to be biopsied. Therefore, a partial resection of the retroperitoneal mass was performed for the purposes of debulking and establishing a histopathological diagnosis. During surgery, a huge, fixed, hard mass with a smooth surface was found around the abdominal aorta; this mass was hypervascular and fixed. A retroperitoneal mass of size 12.2 × 6.3 × 4.5 cm was removed. On gross examination, the retroperitoneal mass weighed 283g and was a pale brown color with a whitish homogeneous cut surface. Microscopically, the resected mass was characterized by spindle cell proliferation. Immunohistochemical (IHC) staining for pancytokeratin (AE1/AE3), CD117, PDGFR, CD34, actin, desmin, and S-100 protein was performed. IHC staining results were positive for actin and desmin (Figure 2). The mitotic activity was < 1 mitosis event per 50 high-power fields (HPFs) with mild to focally moderate nuclear atypia and there was no evidence of tumor cell necrosis. The final pathological diagnosis was a retroperitoneal STUMP tumor, with a recommendation for careful clinical follow-up. Additional IHC staining for Ki-67, p53, estrogen receptor (ER), and progesterone receptor (PR) was performed. The Ki-67 labeling index was less than 5%, and results were negative for p53. IHC staining results for both ER and PR were positive (Figure 2). We considered angioembolization as treatment for the tumors because of the tumor hypervascularity. Angioembolization was performed with polyvinyl alcohol particles (Boston Scientific, Fremont, California, USA) and multiple microcoils (Tornado, Cook, Bloomington, IN, USA) twice in five months. Both the lumbar arteries, the right internal iliac artery and the left renal capsular artery supplying the tumors were embolized. At one month after the last angioembolization, an abdominal CT scan was performed. There was no significant interval change in the size and extent of the retroperitoneal masses.

## Discussion

The major histopathological parameters for assessing the diagnosis and prognosis of smooth muscle tumors are cytological atypia, mitotic index, and coagulative tumor cell necrosis. Based on these parameters, the definition proposed by Bell *et al. *is as follows [3]. Leiomyosarcomas are defined as tumors with at least two of the following three features: diffuse cytological atypia, tumor cell necrosis, and ≥ 10 mitosis events per 10HPFs. Leiomyomas are defined as tumors with no or mild cytological atypia, no tumor cell necrosis, and < 5 mitosis events per 10HPFs. STUMPs are defined as tumors with following features: (1) focal moderate to severe cytological atypia, no tumor cell necrosis, and < 5 mitosis events per 10HPFs, or (2) no or mild cytological atypia, tumor cell necrosis, < 10 mitosis events per 10HPFs.

STUMPs are relatively rare tumors. The data available on clinical characteristics, biological behavior, and follow-up is insufficient, and therefore it is difficult to predict the clinical course of STUMPs. Guntupalli *et al. *reported the follow-up data of 41 patients diagnosed with uterine STUMPs [3]. The mean follow-up time was 45 months (range one to 171 months). Three patients (7.3%) had a recurrence during the follow-up period, one patient presented with a pelvic mass and a pulmonary nodule, and two patients presented with retroperitoneal and pelvic masses. They developed recurrent disease at 13, 47, and 68 months, respectively, after the initial surgical intervention. Berretta *et al. *also presented a report of three cases with STUMPs [1]. During the follow-up, one patient developed diffuse lung metastases nine years after the original diagnosis. The patient was then treated with gonadotropin-releasing hormone agonists and an aromatase inhibitor. Amant *et al. *reported a retroperitoneal/pelvic relapse after four years in a patient diagnosed with a STUMP and treated with hysterectomy and adnexectomy. This patient experienced a relapse of leiomyosarcoma with a malignant evolution of the primary tumor [4]. As mentioned above, previous studies suggest that STUMPs are usually clinically benign, but they should be considered as tumors of low malignant potential because they can occasionally recur or metastasize to distant sites, years after hysterectomy [2]. Therefore, patients diagnosed with STUMPs should receive long-term follow-up.

Our patient also developed a retroperitoneal recurrence with lung metastases, eight years after her hysterectomy. Although we could not confirm the pathology of the previous hysterectomy specimens, the retroperitoneal mass showed a strong positivity for ER and PR. This hormone receptor positivity could be useful to determine whether a retroperitoneal smooth muscle tumor is of uterine type, because the extrauterine soft tissue smooth muscle tumors are generally ER/PR negative [5,6]. Lung metastases were not confirmed by histopathology, but our patient's pulmonary lesions were consistent with typical cavitating metastatic nodules, which had been reported in the previous literature. Additionally, she had no other respiratory symptoms or laboratory findings suggestive of other pulmonary diseases.

There are some studies on the molecular markers or cytological/histological patterns that are predictive of the clinical course of STUMP. Burns *et al. *reported on the importance of coagulative tumor cell necrosis as the single most powerful factor for malignant behavior among morphological features [7]. Some studies have emphasized the importance of Ki-67, as a proliferation marker and p53 overexpression that indicates p53 tumor suppressor gene mutation [8]. Overexpression of p53 and a high Ki-67 labeling index are frequently associated with leiomyosarcoma, and therefore these markers may be useful IHC parameters to distinguish between cases of malignant smooth muscle tumors and those of uncertain or borderline histology. Some studies also have reported that ER/PR-positive tumors showed a benign clinical behavior in contrast with markedly reduced hormone receptor positivity in leiomyosarcoma [4,8].

The lesion in our patient's case showed focal moderate nuclear atypia, but mitosis activity was < 1 mitosis event per 50HPFs without necrosis. Additionally, the lesion was p53 negative with low Ki-67 expression and was ER/PR positive. Therefore, it can be expected to be a STUMP of low malignant potential. In fact, the retroperitoneal mass and metastatic lung nodules showed no interval change in their size over a period of at least five years. However, the multiple lung metastases were an unusual finding that was unlike its benign clinical course.

There is no consensus on the preferred management of benign metastasizing leiomyoma or STUMP with inoperable metastatic lesions. Some studies support the hormonal dependency of uterine smooth muscle tumor, and have revealed the effects of medical castration (that is, suppression of the production of estrogen) [9,10]. The role of chemotherapy in these tumors with a benign nature remains uncertain. We tried angioembolization in the treatment of our patient's inoperable retroperitoneal STUMP, since the tumor showed hypervascularity. However, there was no decrease in the size of the tumors on a follow-up CT scan. We hypothesized that angioembolization was less effective in reducing tumor masses in our patient due to multiple, numerous, minute feeding arteries from both the intra-peritoneal and retroperitoneal branches of the abdominal aorta and both the iliac arteries.

## Conclusion

STUMPs have an unpredictable clinical course. On the basis of our experience and review of the literature, relapses appear to occur generally after a long disease-free interval of up to several years. Therefore, in the patients with STUMPs, a long-term, close follow-up is required. The focus of future research should be on finding the markers based on better understanding of the molecular pathways leading to malignant transformation, which will allow us to predict the clinical behavior of these tumors of uncertain malignant potential.

## Consent

Written informed consent was obtained from the patient for publication of this case report and any accompanying images. A copy of the written consent is available for review by the Editor-in-Chief of this journal.

## Competing interests

The authors declare that they have no competing interests.

## Authors' contributions

HSW drafted the manuscript. HSW and HGC collected clinical data and performed the literature review. KJL evaluated hematoxylin and eosin and immunostained slides. All authors read and approved the final manuscript.
